# Situation, challenges, and SEOM recommendations for the future of undergraduate education in Oncology in Spain

**DOI:** 10.1007/s12094-019-02230-8

**Published:** 2019-11-08

**Authors:** M. A. Segui, J. J. Cruz, E. Alba, J. Feliu, C. Jara, F. Rivera, A. Rodriguez Lescure, A. Lorenzo, M. Martin

**Affiliations:** 1 Medical Oncology Department, Parc Tauli Hospital Universitari, Universitat Autònoma de Barcelona, 08208 Sabadell, Spain; 2 Medical Oncology Department, Hospital Clínico Universitario, Universidad de Salamanca, Salamanca, Spain; 3 Medical Oncology Department, Hospital Universitario Virgen de La Victoria, Málaga, Spain; 4 Medical Oncology Department, Hospital Universitario la Paz, Universidad Autónoma de Madrid, CIBERONC, Madrid, Spain; 5 Medical Oncology Department, Hospital Universitario Fundación Alcorcón, Universidad Rey Juan Carlos, Madrid, Spain; 6 Medical Oncology Department, Hospital Universitario Marqués de Valdecilla, Santander, Spain; 7 Medical Oncology Department, Hospital General Universitario de Elche, Elche, Spain; 8 Medical Oncology Department, Hospital Universitario de Puerto Real, Cádiz, Spain; 9 Medical Oncology Department, Hospital General Universitario Gregorio Marañón, Instituto de Investigación Sanitaria Gregorio Marañón, CIBERONC, GEICAM, Universidad Autónoma de Madrid, Madrid, Spain

**Keywords:** Undergraduate, Oncology, Education, Future, Recommendations, Spain, SEOM

## Abstract

**Purpose:**

The Spanish Society of Medical Oncology (SEOM, for its Spanish acronym) would like to attest to the relevance of training in Oncology as part of the undergraduate education in Medicine program and issue recommendations to improve said training, with the aim of responding better to the challenges that cancer poses to our society.

**Materials and methods:**

The curricula of 42 schools of medicine were reviewed with interviews with at least one teaching medical oncologist from each faculty. The qualitative and opinion analysis was completed by means of an online questionnaire targeting lecturers, resident tutors, and residents in Medical Oncology (MO), enabling the detection of needs and areas for improvement at an organizational level and in terms of skill acquisition.

**Results:**

While the number of medical schools with a specific, mandatory program in MO has grown by up to 90%, it has not been accompanied by an increase in independent programs. Instead, they largely consist of programs shared with other specialties (61% of the medical faculties). In most of the undergraduate education programs, Oncology contents are fragmented and approached from the perspective of each organ system.

**Conclusions:**

Despite the positive evolution in recent years, the heterogeneity in Oncology contents during undergraduate education training continues to be remarkable. Cross-sectional programs with an integral vision, taught in the final years of undergraduate medical education would be desirable. Among the recommendations for improvement of training in Medical Oncology, the SEOM proposes that updated, theoretical content be incorporated and clinical practice in Medical Oncology departments be promoted.

## Introduction

Cancer, as one of the great challenges of our society, demands physicians with solid training and skilled to provide the very best response at all levels, from prevention and early detection to the treatment of patients with cancer.

In the past 20 years, the number of tumors diagnosed has undergone constant growth in Spain, due, not only to the growth in population, but also to early detection techniques and to the increase in life expectancy [1].

Improved prevention and early diagnosis, better patient follow up, guaranteed access to the most efficacious treatments for each tumor, the practice of personalized and precision medicine, facilitating the best treatment available depending on the genomic and clinical characteristics of the patient, are mere examples of the challenges the medical oncologist faces. Given the magnitude and relevance of these challenges, different specialists, including Primary Care professionals also face these challenges.

The changes in the epidemiological pattern of the disease, the new evidence, and technological progress will necessarily have an impact on how to perform and organize the work and, therefore, on the training needs and competence-building of future physicians to be able to adequately care for patients with cancer.

Aware of the relevance of teaching Oncology during medical training, different institutions such as the World Health Organization (WHO) and the International Union against Cancer (UICC) have worked on drafting recommendations, from the very inception of the specialty itself in 1978 [2].

In 1989, the European Commission, within the framework of the “Europe against Cancer” initiative, identifies the need to influence training in Oncology during undergraduate and graduate training. In addition, and together with the European Organisation for Research and Treatment of Cancer (EORTC*),* the Commission published the Curriculum in Oncology for Medical Students in Europe [3], which represents a proposed common curriculum in Oncology for medical students in Europe. The proposal was unanimously approved by 50 deans of medical schools from 17 countries, who participated in a joint workshop of the European Commission (EC) and the European Organisation for Research and Treatment of Cancer (EORTC). The proposal sought to respond to the training deficits in Oncology that had been detected and to the growing needs for skills in the areas of prevention, early diagnosis, treatment, and palliative care in the oncology patient.

The proposed European curriculum highlights the need for coordination, through a figure or system of coordination for the Oncology training program, with the aim of preventing omissions in important areas and of guaranteeing a multidisciplinary approach to cancer and contributing to greater homogeneity in patient management. The presence of the oncologist is recommended in the academic field, as is a specific evaluation of knowledge about Oncology.

The SEOM, for its part, from its very creation, has demonstrated its concern and firm commitment to medical students’ training in Oncology, as attested to at the various meetings of instructors held in the years 2006, 2007, 2010, and 2011. At the first meeting, held in Salamanca in 2006, a consensus document was drafted that included considerations regarding the need for an Oncology program; the document was approved at the meeting held in Cordoba in 2010 [4]. The document included a proposal regarding the contents that had been agreed upon with medical school faculty.

In 2013, SEOM drafted the report, “Formación de pregrado en Oncología. Una asignatura pendiente” [Undergraduate training in Oncology: an unresolved issue] [5], which, after reviewing the situation of training in Oncology at the undergraduate level, included a series of recommendations for the organization of Medical Oncology programs in medical schools.

The major challenges faced by Oncology make it particularly necessary that solid training for our future physicians be guaranteed, enhancing training in Oncology with an integral and multidisciplinary view at the undergraduate level. SEOM seeks to contribute to this aim by conducting regular studies of the status of undergraduate training in Oncology and issuing recommendations for the future. SEOM’s proposal includes a global approach to Oncology in the final years of the undergraduate education program that integrates the knowledge acquired in the various system subjects in future physicians’ clinical training so that they will have an integral, cross-sectional, multidisciplinary view of the approach to cancer.

## Material and methods

In order to conduct the project, a working group was designated consisting of members of SEOM with different responsibilities in the field of Medical Oncology training. This group validated the study’s analytical methodology, as well as the conclusions and recommendations included in the study.

The analysis of the status of training in Oncology during undergraduate education in medicine was carried out by analyzing the study plans and teaching guidelines of the subjects that included content regarding Oncology at the 42 medical schools existing in Spain during the 2016–2017 academic year, for the purpose of understanding the current situation and revealing trends in recent years. With this aim of analyzing their evolution, an analytical structure was maintained that was similar to studies performed by SEOM in previous years.

The list of universities that offer undergraduate education in medicine can be consulted at (https://seom.org/adjunt/Facultades_de_Medicina_en_Espanya.pdf). The data presented correspond to 41 medical schools, since only the first 2 years of the undergraduate degree in Medicine are taught at the Huesca School for Health Sciences and Sports (University of Zaragoza); training in Medical Oncology is, therefore, not taught. This preliminary information was validated by means of an interview with a medical oncologist involved in teaching undergraduate education at 33 of the 41 medical schools.

The quantitative analysis was completed through an online questionnaire targeting different profiles, including medical oncologists with teaching responsibilities in undergraduate education in medicine, resident tutors, and residents, in addition to the members of the project working group.

The opinion poll made it possible to collect information about the main deficits identified as regards undergraduate education in Oncology, respondents’ opinion as to the optimal organization of teaching Medical Oncology in the future, type of key competences to be acquired during undergraduate education, and leading deficits perceived by residents in Medical Oncology, in addition to the factors during undergraduate training that had the biggest impact on their decision to specialize in Medical Oncology.

The questionnaires used are available at (General data: https://bit.ly/2TbCssA; Teachers: https://bit.ly/2R5lTwk; OM residents: https://bit.ly/2Czd7Se: Other esidents: https://bit.ly/2HAhVfA).

All told, 65 people responded to the survey, 28 of whom were instructors, resident tutors, and other profiles; the remaining 37 questionnaires were answered by residents.

Finally, based on the results of the analysis, a proposal of recommendations was drafted that was agreed upon during an in-person meeting of the project working group.

## Results

### Diagnosis of the situation of training in Medical Oncology during undergraduate medical education

During the 2016–2017 academic year, 40 universities offered undergraduate medical educations in Spain at 42 schools of medicine (given that the University of Zaragoza and the University of Castilla-La Mancha have two medical schools that offer undergraduate education in Medicine). Eighty percent (33) of these universities are public and 20% (nine) are private.

### Modalities of specific Medical Oncology training programs

**Fig. 1:**
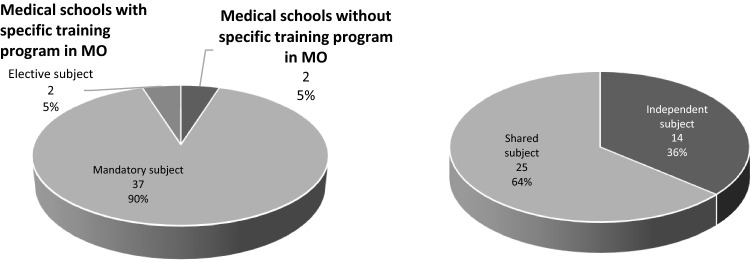
Percentage of medical schools with a specific program in Oncology, with or without an independent subject

Ninety-five percent (95%) of the medical schools have a specific training program in Medical Oncology. In the sphere of this study, specific training program were considered to be those that include a syllabus and well-defined, specific Oncology content. This specific program is taught through a mandatory subject in 90% of the medical schools (Fig. 1).

Only 14 of the 39 specific Medical Oncology programs (36%) structured their content into independent subjects. In the case of subjects in which Oncology shares content with other specialties, the most common situation is that of a subject that shares content about Hematology (54%) and Radiation Oncology (33%).

The various teaching modalities of Medical Oncology at the different medical schools were analyzed, insofar as the subject typology (compulsory or elective), content shared with other specialties, and the number of credits. The most common modality of the specific Medical Oncology curriculum (which corresponds to 39% of the subjects) consists of incorporating Oncology content into a subject that is shared with other specialties, with a dedication of between 3 and 4.5 credits dedicated to Oncology (Table 1).

**Table 1:** Teaching modalities of the specific Medical Oncology program

Modality	No. medical schools	%
Independent compulsory subject with six credits	1	2
Independent compulsory subject with fewer than six credits	5	12
Independent elective subject	2	5
Joint subject with another specialty, with between three and four credits dedicated to Medical Oncology, but with an independent examen	6	15
Joint subject with another specialty, with between three and 4.5 credits	16	39
Joint subject with another specialty and FEWER than three MO credits	9	22
No specific program in Medical Oncology	2	5
Total	41	100

Although during the 2016–2017 academic year, six medical schools had a specific, mandatory Medical Oncology subject, this subject accounted for six credits only at the University of Salamanca School of Medicine.

In most cases, the subjects with Medical Oncology content are taught in the final years of the undergraduate education program. In 51.3% of the cases, the subjects with Medical Oncology content are taught during the final two years of undergraduate education; 35.9%, in the fourth year, and 12.82% of the subjects are taught in the third year.

### Oncology and Palliative care

**Fig. 2:**
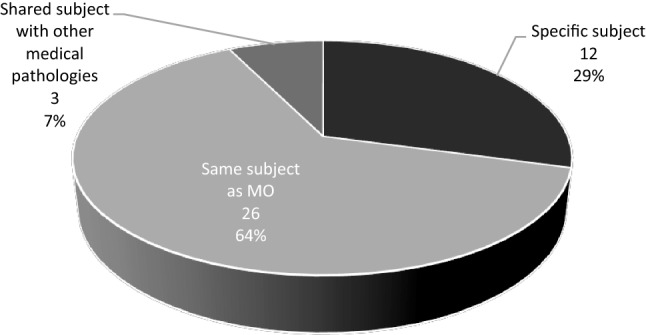
Organization of the contents of palliative care in undergraduate medical education

Content regarding Palliative Care is taught in the same subject as Medical Oncology at 63% of the medical schools. Twelve medical schools (30%) had a specific subject dedicated to Palliative Care. Palliative Care was included in subjects containing other medical pathologies (other than Medical Oncology) at only three medical schools (7%). At five medical schools that have a specific subject dedicated to Palliative Care, content about Medical Oncology is also included in this course (Fig. 2).

### Credits dedicated to Medical Oncology

During the academic year studied, 3.16 was the overall average of credits dedicated to Medical Oncology. In the independent subjects, the average number of credits was slightly higher − 3.81. In the subjects shared with other specialties, the average number of credits dedicated to Medical Oncology was 2.96 (Table 2).

**Table 2:** Average number of credits dedicated to Medical Oncology according to the type of subject

	Average credits overall subject	Average credits in MO*
Independent subjects	3.81	3.81
Joint subjects	7.21	2.96
Overall	6.51	3.16

*Estimated according to the hours of teaching dedicated to Medical Oncology

### Participation of Medical Oncologists in teaching undergraduate programs in Medicine

A medical oncologist is involved in teaching at 90% of the medical schools. Within the framework of this project, a total of 139 oncologists have been identified as teaching subjects with Medical Oncology content. On average, this figure accounts for 3.56 instructors who are specialists in Medical Oncology at each medical school. Taking into account a total of 1216 clinical Medical Oncologists in Spain, as estimated in the latest census performed by SEOM, corresponding to the year 2014 in Spain [6], only 11.43% would be involved in teaching undergraduate education in medicine.

As for distribution by teaching position, of the 139 medical oncologists, only eight are department chairs and 15 are full professors.

A medical oncologist participates in the coordination of the subject, either exclusively or together with faculty members from other specialties in 59% of the subjects with specific medical oncology content. In the case of subjects in which there is no medical oncologist involved in coordination, more often than not, the subject is coordinated by a hematologist (46%) or radiation oncologist (23%).

### Theoretical and practical teaching

**Fig. 3:**
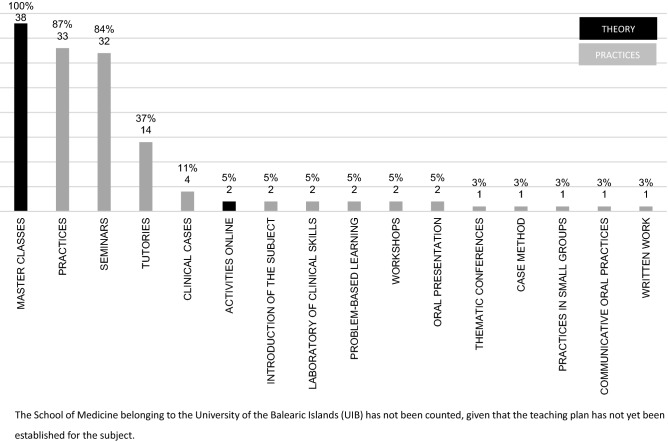
Percentage of subjects with different training modalities

Teaching continues to be in the form of master classes in most cases in practically all of the subjects. Different types of practica and seminars are also usual with greater or lesser theoretical or practical content (Fig. 3). A classification of teaching activities was made on the basis of their theoretical or practical nature. Based on this distribution, the ratio of theoretical: practical hours is 1:0.8 h in the case of subjects shared with other specialties and slightly greater, 1:0.94 h, in independent subjects.

Sixty-nine percent (69%) of the subjects with Medical Oncology content offer clinical practice in a medical oncology service, with a mean of 22.67 h (Table 3).

**Table 3:** Subjects with Medical Oncology content that offer the option of carrying out clinical practice on a Medical Oncology service

Practicum on Medical Oncology service	No. of subjects	%
Yes	27	69
Possible	3	8
No	6	15
Lacking information	3	8
Total	39	100

At practically all the universities, there is the possibility of participating in clinical practica in the Medical Oncology Department as part of the clinical rotations during the fifth or sixth year.

In total, 158 centers have been identified as being associated to or collaborating with medical schools in Spain. Of them, 98 have been identified as having a medical oncology service, which means that a mean of 2.33 medical oncology services linked to each school of medicine. This ratio is 2.44, in the case of public medical schools, and slightly higher, 2.89, in the case of private medical schools, bearing in mind that there are some medical oncology services that are involved in teaching at more than one.

Of the services that participate in the clinical practicum of subjects with medical oncology content analyzed, all the medical oncologists belonging to the service are involved in its teaching at only 48% of the cases.

### Systems of evaluation

**Fig. 4:**
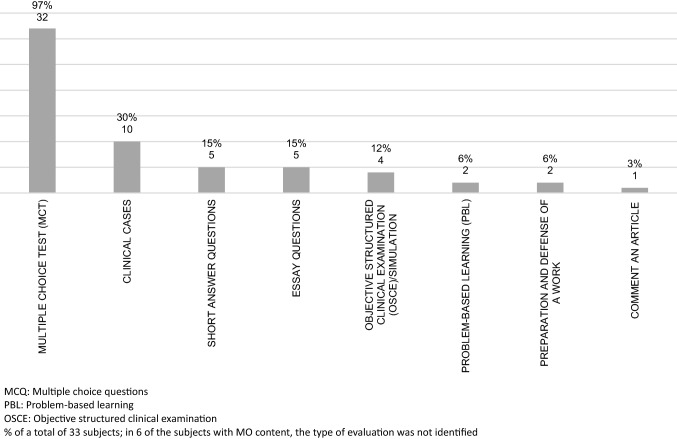
Evaluation methodology applied in subjects with MO content (%)

The most common system of evaluation for subjects with medical oncology contents continues to be the multiple choice type of exam (97% of the cases), followed by clinical case studies (30%). Short questions and essay questions are likewise commonly used (15%). The most innovative methods of evaluation, such as simulation systems or objective structured clinical evaluation (OSCE), is only applied in 12% of the subjects having medical oncology content (Fig. 4).

Clinical practice is largely evaluated solely on the basis of attendance and direct observation of the student’s participation. In 23% of the cases, a dossier is also created during the practicum. OSCEs are only used in 18% of the cases to evaluate the practicum.

### Comparison with the situation in 2013

Overall, with respect to the analysis conducted for SEOM’s 2013 report, “Formación de pregrado en Oncología. Una asignatura pendiente”, the number of medical schools with a specific, mandatory program in Medical Oncology has risen (Table 4). Nonetheless, this increase has not gone hand-in-hand with an increment in independent programs with most of the programs being common to other specialties.

**Table 4:** Comparison between the 2013 analysis of the “Formación de pregrado en Oncología Una asignatura pendiente” and the current analysis

Criterium	2003	2013	2013 Recommendation	2016
Specific, independent, compulsory program	33.33%	41.66%	Specific, distinct curriculum for Medical Oncology. Core/ mandatory subject	29%
Specific, non-independent, compulsory program	12.50%	25%		61%
Specific, compulsory program	45.83%	66.66%		90%
Specific, independent, elective program	29%	13.88%		5%
Total credits (mean)	Five cred. LOU	3.5 ECTS	Six ECTS	3.2 ECTS
Academic year for the 5–6 years program/3 years educational program	5th or 6th	4th, 5th, or 6th	5th or 6th	3th, 4th, 5th, or 6th
Medical Oncology instructor	40%	69%	Promote instructors having a specific profile and clinical dedication to Oncology	90%
Independent examination	66%	72%	Independent grading	34%

The greater number of medical schools has been accompanied by more professors in Medical Oncology versus previous studies. The trend is toward maintaining a joint exam with the other specialties with which a subject is shared. Given the tendency toward teaching oncology content together with other specialties, the percentage of medical schools at which a final evaluation has decreased and grading is performed independently.

The mean number of credits dedicated to Medical Oncology has remained relatively constant or slightly lower from 3.5 according to the 2013 study to 3.2 ECTS credits on average in 2016. Be that as it may, it has remained far from the recommended 6 ECTS dedicated to medical oncology recorded in the 2013 report.

Due, at least in part, to the decrease in theoretical content in the 6th year in the new study plans, an increase is observed in the programs that include Medical Oncology in 3rd and 4th years.

### Results of the “Situation of the teaching of Oncology in undergraduate education in Medicine” opinion poll

Ninety-two percent (92%) of the faculty who participated in the online poll felt that teaching oncology at the undergraduate level exhibits areas for improvement. The main deficits related with the insufficient number of credits dedicated to Medical Oncology, teaching content in an uncoordinated way, the lack of systems of evaluation that suit the practical competences involved, and the excessive weight of theoretical content in the evaluation.

The instructors who participated in this poll felt that Medical Oncology should be taught as a subject that is integrated or coordinated with other areas of medical pathology, mandatory in the final years of undergraduate training, and endowed with sufficient credits.

As regards the distribution of hours dedicated to theory and practice, most of the instructors agreed that the number of hours of learning by practice should lean toward being equal to the number of hours dedicated to theoretical content.

A fundamental element to enhancing training in oncology during undergraduate education in medicine, the subject must include updated theoretical content and make it possible to perform clinical practice in medical oncology services. The main impediment to implementing an oncology training program perceived by the faculty polled is the current inertia and inflexibility of university structures at all levels, including personnel, subjects, or departments.

Sixty-two percent (62%) of the respondent believe that basic competences are not adequately collected in the training programs. The primary shortcoming in competences among undergraduates is the lack of the fundamental concepts of management of the oncological patient (symptomatology, toxicities, support treatment, etc.), and patient-related competences as to communication skills and the need to reinforce the acquisition skills in communicating with the patient, increasing content about the molecular biology of cancer, and regarding research methodology.

In the opinion of the residents polled, the credits dedicated to Medical Oncology during undergraduate training were insufficient and, in particular, they detected gaps in knowledge about the treatment and management of complications.

### SEOM’s recommendations to improve undergraduate training in Oncology in Spain

As a result of the analysis and the opinion poll conducted, the project working group put forth four recommendations to contribute to medical school graduates acquiring the necessary competences in medical oncology to practice the profession with the utmost guarantees of quality (Table 5).

**Table 5:** SEOM’s recommendations for the organization of Medical Oncology programs

**Recommendation 1: implement a specific program dedicated to Oncology in all undergraduate medical education programs with an integrative, cross-sectional view of the subject, including a specific evaluation** Program integrated in a mandatory subject Having at least 4–6 specific ECTS, depending upon whether palliative care content is included In a subject taught in the last years of the undergraduate education program, preferably in the 5th year, when the student already has a general view of the disease Having an instructor who is a medical oncologist in 100% of the cases, who imparts knowledge about the clinical approach to the patient in routine practice (symptomatology, toxicities, support treatment, etc.) and contributes an integral view of cancer treatment Having aims specifics for the training of competences appropriate to primary care physicians and future specialists in different diseases having greater contact with patients with cancer, avoiding an excessive workload of highly specialized knowledge Coordinated with the content about cancer in other Medical Pathology and Surgical Pathology subjects, thereby preventing redundancies or commissions Reinforce the importance of the clinical content of Oncology, with a specific system and the aim of evaluating competences With the option of practical in Medical Oncology services in 100% of the subjects or rotations
**Recommendation 2:** promote undergraduate student involvement in practica out-patient and in-patient activity, contact with the patient in follow up, long-term survivor, on tumor boards, genetic counseling, and research units, in MO services
**Recommendation 3:** promote content aimed at training for use in clinical practice, avoiding the overload of knowledge specific to an Oncology super specialist. Promote the incorporation of content regarding innovative diagnostic and treatment procedures, including their perspective of evolution in the future
**Recommendation 4:** SEOM believes that the Medical Oncologist must play a relevant role in teaching Medical Oncology at the undergraduate level of education. SEOM will dedicate special effort to promoting Medical Oncologists’ participation in the teaching of undergraduate education, as well as in facilitating Medical Oncologists’ access to accreditation by ANECA as instructors

Implementing a specific program dedicated to Oncology in all undergraduate training in medical programs with an integrative, cross-sectional view of the subject, including a specific evaluation is the leading recommendation. In light of the data regarding the situation in the 2016–2017 academic year, the project's working group felt the proposal made by SEOM at their meeting of faculty in 2010 for a Medical Oncology program to be totally valid and adaptable (Table 6).

**Table 6:** Program of Medical Oncology proposed by SEOM

Medical Oncology	
KNOW	
Recognize, diagnose, and guide management	Only know about
1. Tumor disease: Gnoseologic diagnosis and diagnosis of extension. Prognostic factors and staging factors	1. Cell and molecular biology of cancer
2. Epidemiology of cancer and risk factors	2. Carcinogenesis
3. Primary and secondary prevention	3. Growth of the tumor cell
4. Hereditary cancer and genetic counseling	4. Mechanisms of tumor invasion and metastasis
5. Acute tumor complications: superior vena cava syndrome. Spinal cord compression syndrome. Intracranial hypertension syndrome. Hypercalcemia	
6. Paraneoplastic syndromes (endocrine, neurological, hematological, dermatological, osteoarticular, and other manifestations)	
7. Systemic treatment for cancer: chemotherapy	
8. Systemic treatment for cancer: hormonotherapy	
9. Systemic treatment for cancer: immunotherapy and biological therapies	
10. Evaluation of response to treatment and effects on quality of life (e.g., RECIST, WHO). Clinical trials in Oncology	
11. Acute toxicity of antineoplastic treatment	
12. Support treatment for the patient with neoplasia: general concepts	
13. Support treatment for the patient with neoplasia. Infections in the patient with cancer	
14. Support treatment for the patient with neoplasia. Pain treatment	
15. Support treatment for the patient with cancer. Anemia. Cachexia	
16. Control of syndromes in terminal disease. Sedation	
17. Lung cancer. Natural history, prognostic factors, staging, and treatment strategy	
18. Breast cancer. Natural history, prognostic factors, staging, and treatment strategy	
19. Cancer of the stomach, pancreas, and bile ducts. Natural history, prognostic factors, staging, and treatment strategy	
20. Colorectal cancer. Natural history, prognostic factors, staging, and treatment strategy	
21. Ovarian cancer. Natural history, prognostic factors, staging, and treatment strategy	
22. Head and neck cancer. Natural history, prognostic factors, staging, and treatment strategy	
23. Prostate cancer. Natural history, prognostic factors, staging, and treatment strategy	
24. Bladder, urinary tract, and kidney cancers. Natural history, prognostic factors, staging, and treatment strategy	
25. Germ cell tumors. Natural history, prognostic factors, staging, and treatment strategy	
26. Cancer of the cervix and endometrium. Natural history, prognostic factors, staging, and treatment strategy	
27. Sarcomas. Natural history, prognostic factors, staging, and treatment strategy	
28. Melanomas. Natural history, prognostic factors, staging, and treatment strategy	
29. Tumors of the central nervous system. Natural history, prognostic factors, staging, and treatment strategy	
30. Cancer of unknown origin. Natural history, prognostic factors, staging, and treatment strategy	

Medical Oncology		
Know how to do		
Know how to do competently (routinely and without supervision)	Have practiced with supervision (under the tutor’s supervision)	Have seen it practiced by an expert
1. Clinical history oriented toward cancer	1. General management of tumor syndromes	1. Interventional procedures in the diagnosis and treatment of cancer patients
2. By means of physical examination, recognize the existence of the most common tumors and their complications, oriented towards their natural history	2. Management of the most common tumors and their complications	
3. Indicate and interpret complementary testing to diagnose the nature and extension of different tumors		
4. Indicate early detection and screening procedures		
5. Indicate diagnostic procedures for tumors when facing syndromes and warning signs		

The remaining recommendations include promoting undergraduate student participation in the activity of the Medical Oncology services, the incorporation of content about innovative diagnostic and treatment procedures and their perspective looking toward the future. Finally, at SEOM we support the role of the Medical Oncologist in teaching undergraduate education and recommend promoting actions that make it easier for physicians to develop teaching careers.

## Discussion

Aware of the importance of undergraduate education in Oncology, SEOM has proven its commitment to the improvement of said training by organizing meetings and monographic fora, as well as putting forth proposals and recommendations for specific Medical Oncology programs to be carried out.

Royal Decree 1393/2007, dated 29 October, which provides for the organization and planning of official university education [7] made it possible to develop the procedure by which to design and establish requirements for university curricula. By virtue of the [principle of] university autonomy, the universities themselves are responsible for creating and proposing the education to be taught and the degrees to be issued and, with the aim of promoting curricular diversification, the organization of university education is made more flexible.

Study plans are understood as a project of implementation for university education, for the purpose of students’ acquiring competences. This same Royal Decree establishes the verification system for the study plans that are elaborated by the universities, through the University Governing Board and authorized by the corresponding Autonomous Community. Their evaluation is the responsibility of the assessment agencies (the National Quality Assessment and Accreditation Agency of Spain (ANECA for its Spanish acronym) or the evaluation bodies determined in the Law regarding Autonomous Communities).

In the Clinical Practice in Humans training module, Order ECI/ 332/ 2008, dated 13 February, that sets forth the requirements that must be fulfilled to verify official university degrees, that determine that a person can practice medicine [8], reflects two skills that are directly linked with Oncology: understand tumor disease, their diagnosis and management, and palliative medicine. Moreover, the official university degrees corresponding to undergraduate education, consisting of 360 credits, as is the case of undergraduate education in Medicine, must renew their accreditation within a maximum period of 8 years from the date of degree verification or from the date of their latest accreditation. When evaluating the proposed study plans for degrees that have been designed, ANECA has confirmed that they are in line with the European Higher Education Area and that the results of the degree are adequate and enable the students to acquire the skills established.

On the basis of the review carried out, providing data regarding the situation during the 2016–2017 academic year, the project working group proposes a series of recommendations aimed at improving undergraduate education in Oncology.

The first one is the implementation of a specific program dedicated to Oncology in all undergraduate medical degree programs. After reviewing the proposal that was agreed upon at the meeting of SEOM instructors in 2010 to design Medical Oncology teaching programs, the project working group deemed it fully valid and adaptable to different realities. The competences defined insofar as knowing and knowing how are concerned can serve as a basis for both designing programs, as well as in the processes of accreditation and reaccreditation of undergraduate education programs. Likewise, they are considered to be an adequate foundation upon which to define student evaluation systems, in different modalities, including innovative methodologies, such as ECOEs.

Consequently, it is regarded as being a flexible and adaptable proposal that, in addressing the autonomy of the university, responds to the evolution of the specialty, inasmuch as there are more and more tumors for which medical treatment is key and in practically all the cases, integral or multidisciplinary treatment is essential.

With respect to its implementation and in light of the results of the analysis and the evolution experienced since 2013, the working group identified a series of characteristics it deems fundamental with respect to the obligation, structure, and minimum time dedicated to the program, so as to guarantee the minimum quality in keeping with the relevance of the pathology.

Finally, the timing for initiating the formalities to renew the accreditation of undergraduate medical degrees by ANECA is identified as being an outstanding opportunity to revise how Medical Oncology programs are organized in undergraduate educations of medicine.

## Conclusions

Training in Medical Oncology still has a long way to go to become an independent, compulsory subject of at least four ECTS taught by a medical oncologist at all medical schools, which have a growing presence at said medical schools. The evaluation model continues to be linked to the lack of independence from other subjects; hence, few medical schools evaluate it as an independent element. Training activities that include clinical practice at all levels and stages of the care process must still be promoted, as well as elements that incorporate the perspective of integral, innovative treatment of the future.
